# Supplementary material for: Transcriptomic and Metabolomic Analyses Provide Insights into the Pathogenic Mechanism of the Rice False Smut Pathogen Ustilaginoidea virens
Source: Int J Mol Sci. 2023 Jun 28;24(13):10805. doi: 10.3390/ijms241310805 (PMC10342015; doi:10.3390/ijms241310805)
Supplement: Supplementary file 1 [file ijms-24-10805-s001.zip › Supplementary Figures S1-S3.pdf]

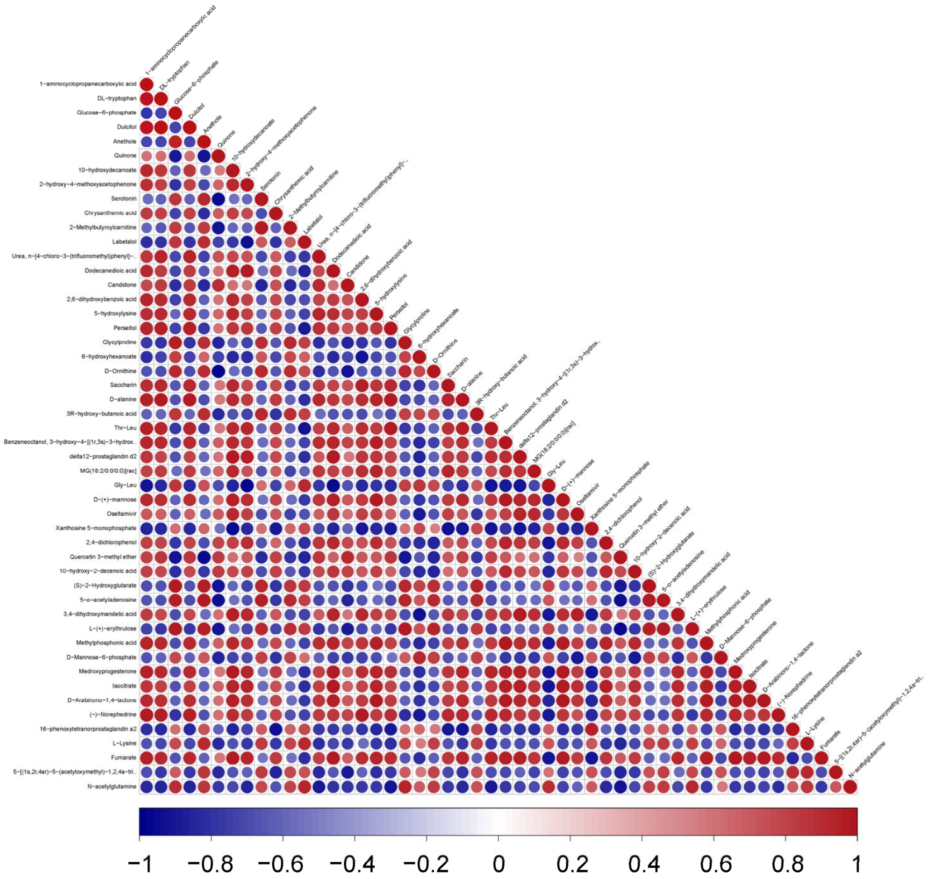
1-aminocyclopropanecarboxylic acid
DL-tryptophan
Glucose-6-phosphate
Dulcitol
Anethole
Quinone
10-hydroxydecanoate
2-hydroxy-4-methoxyacetophenone
Serotonin
Chrysanthemic acid
2-Methylbutyroylcarnitine
Labetalol
Urea, n-[4-chloro-3-(trifluoromethyl)phenyl]-..
Dodecanedioic acid
Candidone
2,6-dihydroxybenzoic acid
5-hydroxylysine
Perseitol
Glycylproline
6-hydroxyhexanoate
D-Ornithine
Saccharin
D-alanine
3R-hydroxy-butanoic acid
Thr-Leu
Benzeneoctanol, 3-hydroxy-4-[(1r,3s)-3-hydrox..
delta12-prostaglandin d2
MG(18:2/0:0/0:0)[rac]
Gly-Leu
D-(+)-mannose
Oseltamivir
Xanthosine 5-monophosphate
2,4-dichlorophenol
Quercetin 3-methyl ether
10-hydroxy-2-decenoic acid
(S)-2-Hydroxyglutarate
5-o-acetyladenosine
3,4-dihydroxymandelic acid
L-(+)-erythrulose
Methylphosphonic acid
D-Mannose-6-phosphate
Medroxyprogesterone
Isocitrate
D-Arabinono-1,4-lactone
(-)-Norephedrine
16-phenoxytetranorprostaglandin a2
L-Lysine
Fumarate
5-[(1s,2r,4ar)-5-(acetyloxymethyl)-1,2,4a-tri..
N-acetylglutamine
−1
−0.8
−0.6
−0.4
−0.2
0
0.2
0.4
0.6
0.8
1

(**A**)

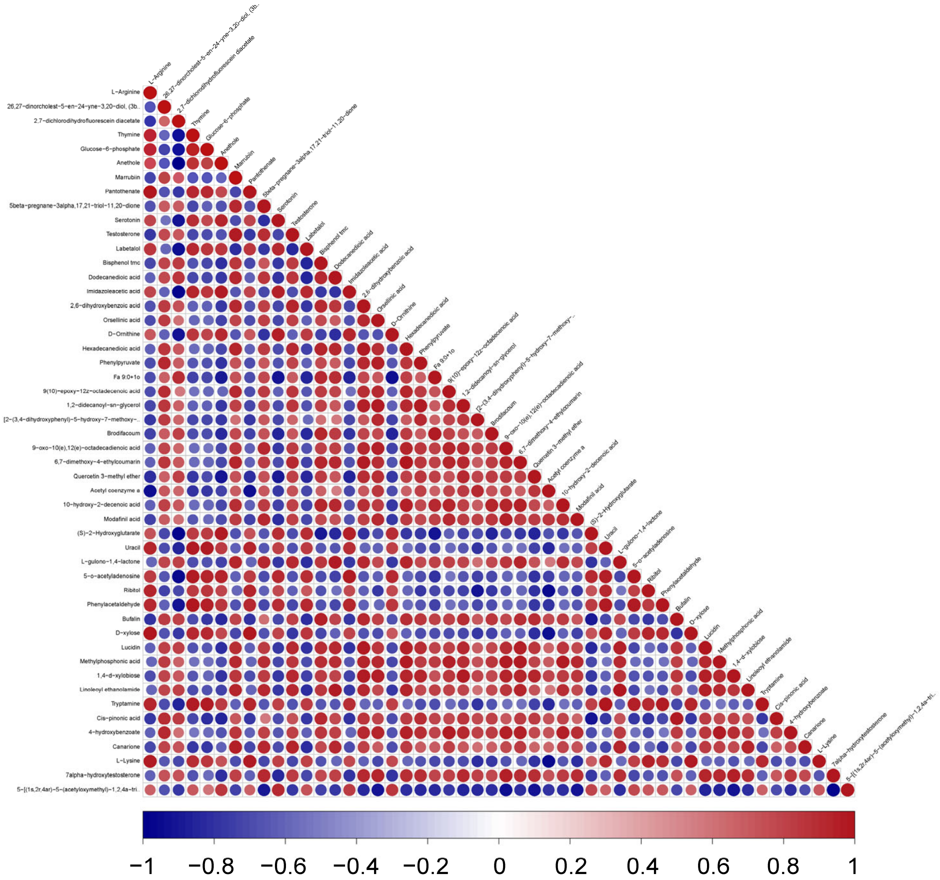

(**B**)

Figure S1. Correlation chart of the top 50 different metabolites. **A**. S1 vs. S0. **B**. W1 vs. W0. Note: The red shape indicates a positive correlation and the blue shape indicates a negative correlation.

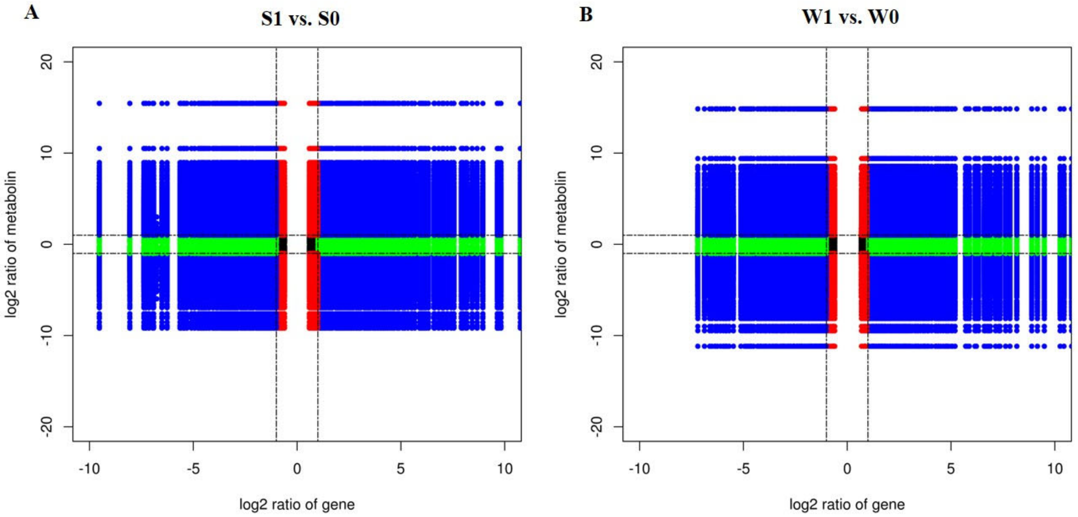

Figure S2. Nine-quadrant diagram of gene and metabolite correlation analysis. **A**. S1 vs. S0. **B**. W1 vs. W0. Note: In quadrant five, the gene and metabolite were not differentially expressed; in quadrants three and seven, the differential expression pattern of genes and metabolites was consistent; in quadrants one and nine, the differentially expressed patterns of genes and metabolites were opposite.

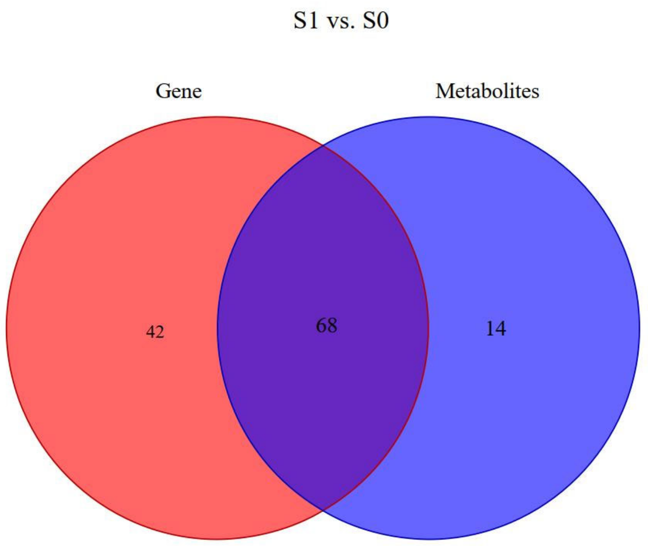
S1 vs. S0
Gene
Metabolites
42
68
14

(**A**)

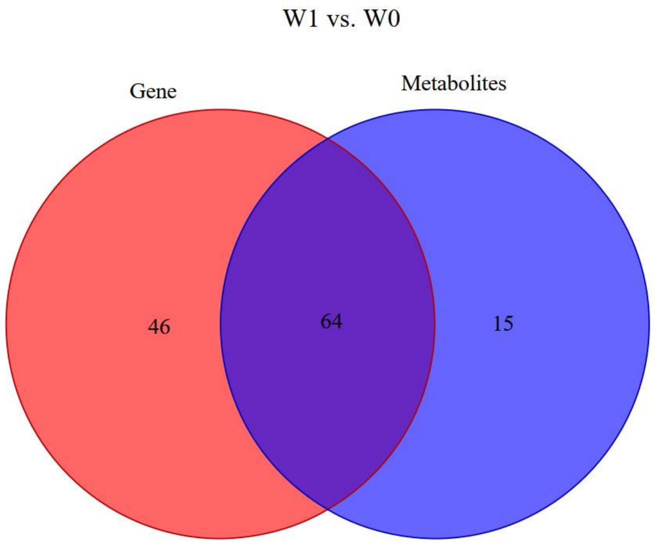
W1 vs. W0
Gene
Metabolites
46
64
15

(**B**)

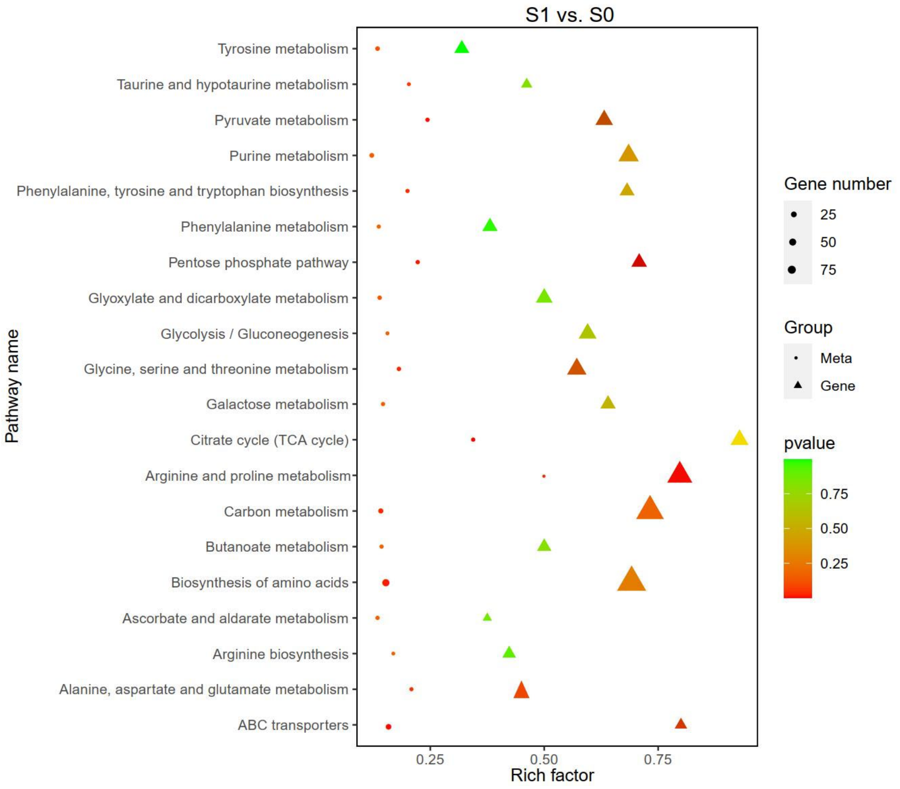

(**C**)

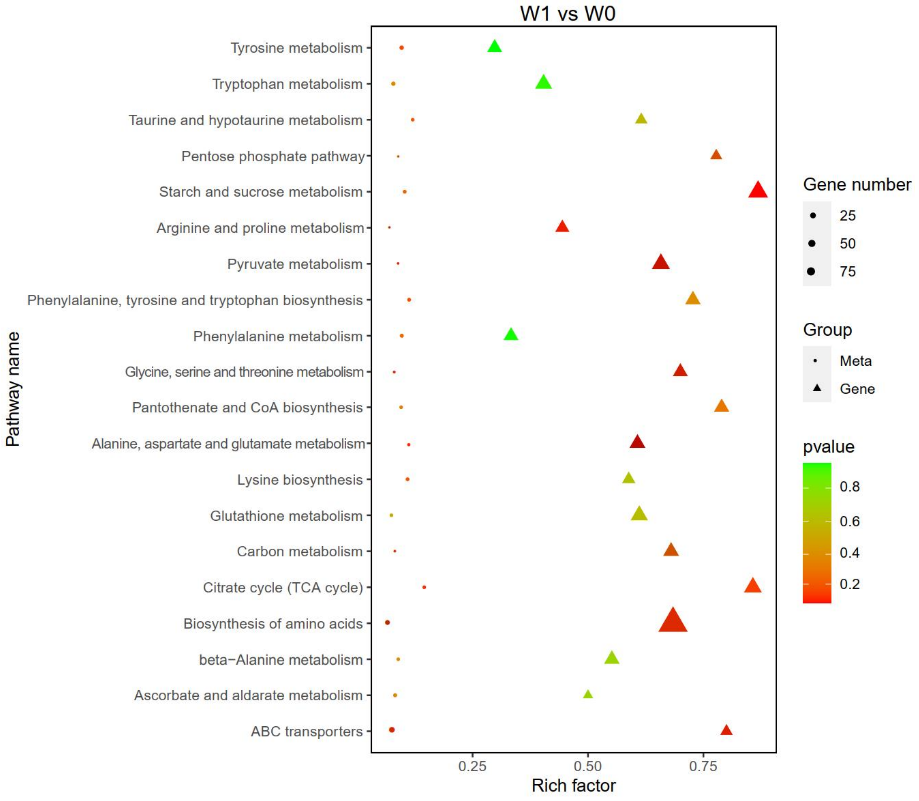

(**D**)

Figure S3. KEGG enrichment analysis of differentially expressed genes and differentially accumulated metabolites. **A-B**. Venn diagram of KEGG enrichment analysis in S1 vs. S0 and W1 vs. W0. **C-D**. Bubble diagram of KEGG enrichment analysis in S1 vs. S0 and W1 vs. W0.
